# Nocardia in an Immunocompetent Host Masquerading As Lung Cancer: A Case Report

**DOI:** 10.7759/cureus.27039

**Published:** 2022-07-19

**Authors:** Jagat B Mahat, Siham Hussien, Rajan M Negassa, Yash Reddy, Girma M Ayele, Miriam B Michael

**Affiliations:** 1 Medicine, University of Maryland School of Medicine, Baltimore, USA; 2 Internal Medicine, University of Maryland Midtown Campus, Baltimore, USA; 3 Internal Medicine, Howard University College of Medicine, Washington, USA; 4 Internal Medicine, University of South Florida, Florida, USA; 5 Internal Medicine, Howard University Hospital, Washington, USA; 6 Internal Medicine, Howard University, Washington, USA; 7 Internal Medicine, University of Maryland, Baltimore, USA

**Keywords:** nocardia in immunocompetent, nocardia as lung cancer, immunocompetent, nocardia, pulmonary nocardiosis

## Abstract

Nocardiosis is generally regarded as an opportunistic infection that can present as a cutaneous, pulmonary, or disseminated disease based on host immunity status. Pulmonary nocardiosis is typically seen in immunocompromised patients; however, it can rarely be present in immunocompetent patients. We present a rare case of an immunocompetent patient who was thought to have a lung malignancy but was found to have pulmonary nocardiosis upon further investigation.

## Introduction

Nocardia spp. is gram-positive, aerobic, and weakly acid-fast intracellular bacteria of low virulence found worldwide in soil and most frequently causes opportunistic infection in immunocompromised hosts and usually self-limited infection in immunocompetent individuals. Nocardia is commonly introduced through the respiratory tract and can progress to a chronic process mimicking tuberculosis, mycotic lung infection, or malignancy [1,2]. We present a case of pulmonary nocardiosis mimicking lung malignancy in a 74-year-old immunocompetent female patient with chronic obstructive pulmonary disease (COPD) and no known history of corticosteroid therapy.

## Case presentation

A 74-year-old female with a history of COPD on 2 liters/minute (L/min) of home oxygen via nasal cannula presented with several weeks of fatigue, subjective fever, and chills. She also had a cough productive of blood-streaked sputum and unintentional weight loss. She was hospitalized four times in the last four months for recurrent pneumonia. The most recent hospitalization showed a positive test for influenza A, along with the imaging finding consistent with multifocal pneumonia. At that point, she received cefepime and oseltamivir phosphate.

 On initial presentation, she was afebrile with tachycardia to 105 beats/minute, with a respiratory rate of 16 breaths/minute, and oxygen saturation of 88% on 2L oxygen via a nasal cannula. On physical examination, the patient was cachectic weighing 39.5 kg (87 pounds), and with fine crackles on the left lung base on auscultation. Initial laboratory results showed leukocytosis with a white blood cell (WBC) count of 14,000/µL and all other laboratory findings were within normal limits. She was treated for a total of six months' duration.

**Figure 1:**
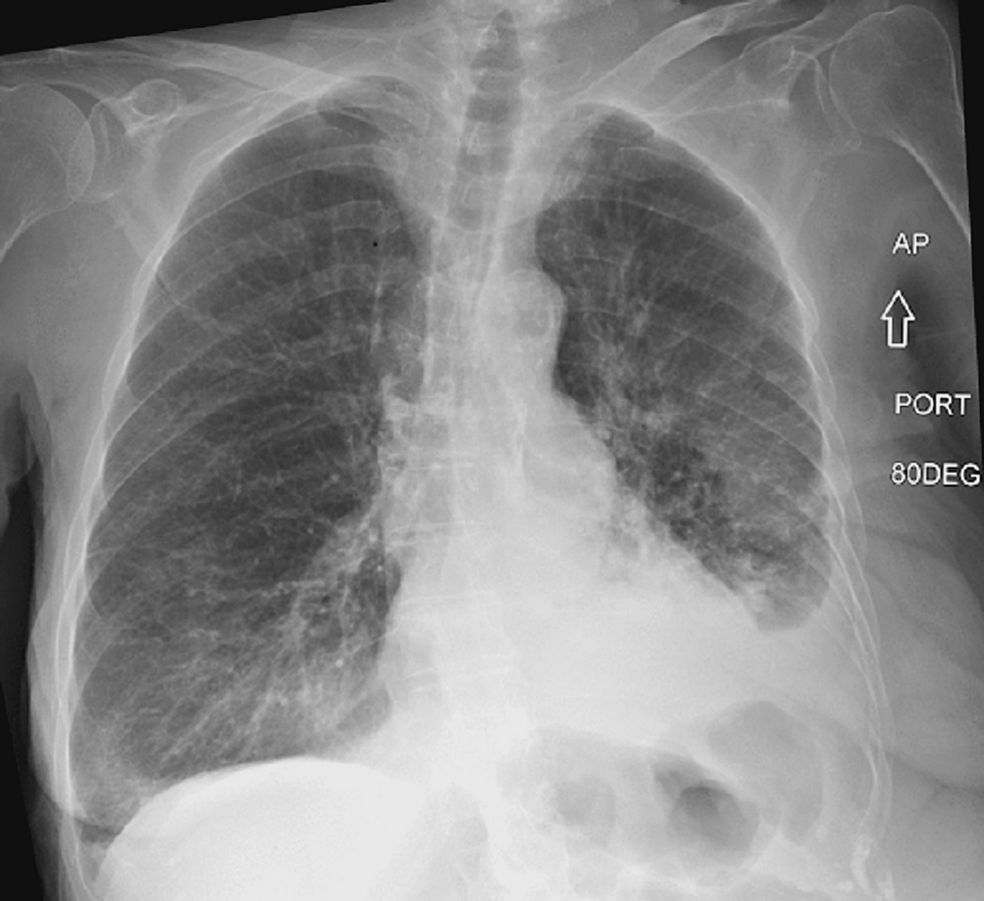
Chest x-ray showing haziness/infiltration on the left lung base along with left pleural effusion.

**Figure 2:**
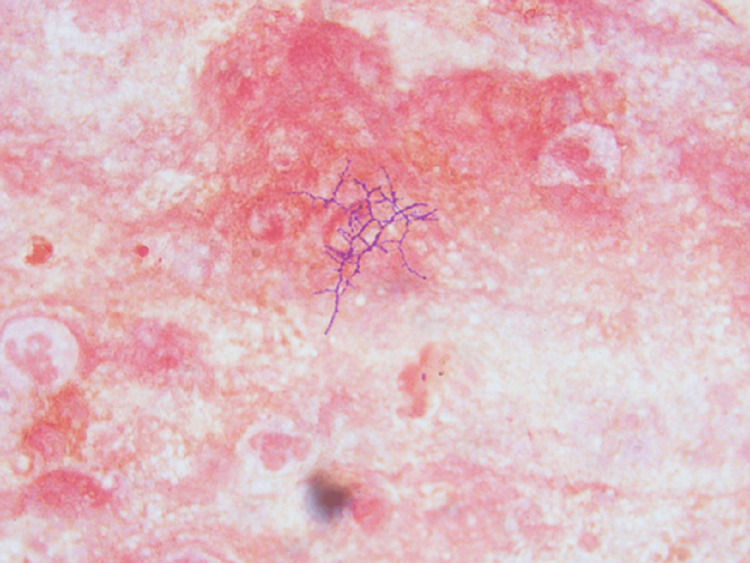
Gram-positive branching filamentous bacteria (Nocardia) seen in sputum culture.

Chest x-ray showed consolidation on the left base, suggestive of possible pneumonia along with left pleural effusion (Figure 1). A diagnostic thoracentesis was done. Pleural fluid analysis done was non-hemorrhagic and was analyzed (findings are outlined in Table 1). Computed tomography (CT) of the chest showed a large infiltrating mass on the left lower lobe and poorly defined nodules in the right lung (unfortunately the films were lost in the system). There was a high suspicion of lung malignancy given the multiple risk factors, including significant smoking history, old age, weight loss, as well as imaging findings. Flexible bronchoscopy and needle biopsy were planned. However, the sputum culture report returned positive for Nocardia (Figure 2). With the culture result, the imaging was reviewed again, and the findings were read as consistent with Nocardia pneumonia. The test for HIV was negative, and a head CT done showed no brain involvement of Nocardia. The patient was started on intravenous trimethoprim-sulfamethoxazole for possible Nocardia pneumonia for six months. The patient showed significant clinical improvement and was discharged to a rehab facility with follow-up, where she continued to have clinical improvement.

**Table 1:** Pleural fluid analysis

Test done	Result
Protein	1.6 g/dL
Lactic Acid Dehydrogenase (LDH)	71 units/liter
White Blood Cell Count	532/hpf
Neutrophils	44%
Lymphocyte	7%
Monocyte	17%
Macrophage	32%
Red Blood Cell Count	254/hpf

 

## Discussion

Pulmonary nocardiosis has been shown to mostly affect immunocompromised patients, particularly those exposed to immunosuppressant agents and high-dose corticosteroid therapies [2]. The weakened respiratory immune defense systems enable infection and colonization of Nocardia in the respiratory tract [3]. Although rare, pulmonary nocardiosis clinical infection in an immunocompetent host is usually in the setting of preexisting structural abnormalities of the lung such as COPD, which causes respiratory, and immune defense system dysfunction and can facilitate lower respiratory tract infections and airway colonization. Furthermore, bacterial colonization on the bronchus alters ciliary motility and causes epithelial damage, facilitating nocardial infection [4].

In immunocompetent patients, the clinical presentation of pulmonary nocardiosis is nonspecific and variable. Common clinical presentations of immunocompetent patients with pulmonary nocardiosis include subjective fever, productive cough with blood-streaked sputum, and unintentional weight loss [5]. In pulmonary nocardiosis, chest radiographic manifestations are pleomorphic and show nodules, masses, and interstitial patterns. CT findings typically show multiple pulmonary nodules, pleural effusion, and chest wall extension [6].

One study identified 30 patients with pulmonary nocardiosis, from the 30 patients 12 0f them (40%) were immunocompetent (IC). The immunocompetent patients were older than the immunocompromised patient and have more underlying lung problems like bronchiectasis and emphysema when compared with the immunocompromised patients. When we see the symptoms both groups presented with the same symptoms as cough (67%), sputum (58%), fever (25%), hemoptysis (25%), and chest pain but the symptoms in immunocompetent were more acute [5].

For these reasons, establishing a diagnosis of pulmonary nocardiosis in immunocompetent patients is often difficult. Without a prior understanding of pulmonary nocardiosis symptomatic, laboratory, and radiographic manifestations, infections may be mistaken for other infections such as lung malignancies, bacterial pneumonia, or tuberculosis, which present similar clinical features. In our case, the diagnosis was made by performing a sputum culture which showed presence of Nocardia. This diagnosis was confirmed by imaging and chest CT findings. This also indicates that invasive diagnostic procedures, like the flexible bronchoscopy and needle biopsy originally planned for our patient, are not essential for pulmonary nocardiosis diagnosis. In all, pulmonary nocardiosis in immunocompetent patients should be included in a differential diagnosis for patients presenting with the aforementioned clinical features.

Treatment of nocardiosis has been sulfonamides since the 1940s, cotrimoxazole (trimethoprim 1 mg/kg/day and sulfamethoxazole 75 mg/kg/day) and some patients even may require surgical drainage. Prolonged therapy is required to prevent relapse, for infection involving the lung treatment should be given for at least six to 12 months and for more than one year for central nervous system involvement [7,8].

## Conclusions

Nocardia is a very rare opportunistic infection that is usually seen in immunocompromised patients. The diagnosis of Nocardia is very difficult in immunocompetent patients because the symptoms and imaging studies are nonspecific. Even though pulmonary nocardiosis is an opportunistic infection, it should be considered in patients without a history of an immunocompromising disease, particularly in COPD patients with frequent exacerbations and presenting with features suggestive of lung malignancy. Thorough clinical evaluation, careful lab investigation, and appropriate clinical correlation of diagnostic imaging findings can assist in making the appropriate and timely diagnosis of Nocardia pneumonia.
